# When Scarcity Meets Sustainability: Consumer Preferences for Recycled Products

**DOI:** 10.3390/bs16050673

**Published:** 2026-04-29

**Authors:** Lei Wang, Yawen Huang, Yunchang Liu, Jiaxin Zhou

**Affiliations:** 1 School of Management, Zhejiang University, Hangzhou 310058, China; ywhuang@zju.edu.cn (Y.H.); 12420017@zju.edu.cn (Y.L.); 2 Neuromanagement Laboratory, Zhejiang University, Hangzhou 310058, China; 12020007@zju.edu.cn; 3 The State Key Laboratory of Brain-Machine Intelligence, Zhejiang University, Hangzhou 310058, China

**Keywords:** resource scarcity, recycled products, holistic thinking, perceived contamination, sustainable consumption, purchase intention

## Abstract

The widespread disposal of waste has led to severe environmental challenges, making the reuse of materials critical for sustainable development. Recycled products, which transform waste into valuable items, are gaining increasing attention from consumers. This research examines how perceived resource scarcity shapes consumer preferences for recycled products and the psychological mechanisms underlying this effect. Across four studies, we induced perceptions of scarcity using two distinct approaches and found that consumers experiencing resource scarcity exhibit higher purchase intentions for recycled products compared with those who do not. This effect is mediated by holistic thinking, which allows consumers to integrate information about a product’s past and present identities, enhancing appreciation for transformation and reuse. Moreover, perceived product contamination moderates this relationship. When contamination concerns are low, scarcity strengthens preference for recycled products, whereas high contamination perceptions weaken or eliminate this effect. These findings extend understanding of how resource scarcity influences sustainable consumption, highlight the cognitive processes driving recycled product demand, and provide practical guidance for policymakers and businesses promoting environmentally responsible consumption.

## 1. Introduction

Resources are essential for human survival and development but are inherently limited due to the imbalance between finite availability and human demand (Roux et al., 2015). As global resource pressures continue to intensify, improving resource efficiency and reducing waste have become increasingly important societal and market priorities (Salciuviene et al., 2022; Syed et al., 2024). Reflecting this trend, firms are increasingly offering reused and recycled products designed to extend product life cycles and make better use of existing resources. Unlike many sustainable products that are primarily valued for their environmental friendliness or energy efficiency, recycled products are distinctive in that they carry a salient past identity, reflecting their association with prior use. This past identity may enhance perceived uniqueness and thereby increase consumer appeal (Kamleitner et al., 2019), but it may also evoke concerns about contamination or impurity (Polyportis et al., 2024). As a result, consumer responses to recycled products are often shaped by both positive sustainability meanings and negative associations tied to their previous life, making them psychologically more complex than many other sustainable goods.

Prior research on recycled product consumption has primarily focused on product characteristics and environmental appeals, showing that consumers’ evaluations and responses are shaped by factors such as perceived quality, contamination concerns, symbolic meaning, and sustainability-related information (Kamleitner et al., 2019; White et al., 2019). However, despite the close conceptual link between recycled products and resource conservation, relatively little research has examined consumer acceptance of recycled products from the perspective of perceived resource conditions. This omission is theoretically important because recycled products are closely tied to the idea of resource reuse, suggesting that consumers’ subjective sense of resource sufficiency or insufficiency may shape how they interpret and evaluate such products.

In this research, we focus on perceived resource scarcity, defined as the subjective perception that one’s available resources are insufficient to meet current needs or goals (Shah et al., 2012; Yang & Zhang, 2022). Prior research has shown that perceived resource scarcity can shape consumer judgment and behavior. Existing scarcity theories have primarily explained these effects in terms of economic trade-offs, narrowed attentional focus, and heightened concern with immediate priorities (Goldsmith et al., 2020). These perspectives help explain many forms of scarcity-driven consumption, especially when consumers respond to price pressure, reduced choice, or short-term constraints. However, they do not fully explain consumer responses to recycled products. Evaluating recycled products involves more than assessing functional utility or economic value. Consumers must also interpret the product’s prior use in relation to its sustainable value and resource-saving implications, while at the same time considering possible contamination concerns. This feature makes recycled products a theoretically distinctive context in which traditional scarcity accounts may be insufficient.

Consistent with this reasoning, prior findings on scarcity and sustainable consumption remain mixed. Some studies suggest that scarcity induces anxiety, reduces self-efficacy, and weakens engagement in sustainable behavior (Park et al., 2020; Saikia & Rahman, 2025; Zhang et al., 2023). Other studies suggest that scarcity heightens sensitivity to resource efficiency and strengthens preference for conservation-oriented options (Mehta & Zhu, 2016; Sachdeva & Zhao, 2021). These divergent findings indicate that the relationship between scarcity and sustainable consumption is not yet well understood, and they leave unresolved the question of how perceived resource scarcity influences consumer responses to recycled products in particular.

To address this question, we propose holistic thinking as an underlying mechanism. Holistic thinking is a cognitive style that emphasizes relationships and the integration of seemingly conflicting attributes (Choi et al., 2007; Nisbett et al., 2001; Wang et al., 2023). We define it here as a mode of thinking that leads consumers to consider how different product meanings fit together rather than evaluating each attribute in isolation. Prior research suggests that perceived resource scarcity can promote more resourceful cognition and more systematic decision making (Mehta & Zhu, 2016). Extending this view, we argue that scarcity may lead consumers to interpret product information in a more relational and context-dependent way. This mechanism is especially relevant because recycled products often combine negative cues associated with prior use with positive meanings related to sustainability and resource value. Under perceived resource scarcity, consumers may therefore be more likely to reconcile these competing meanings within a coherent interpretation, which in turn increases their preference for recycled products.

At the same time, this effect may depend on perceived product contamination. In this study, perceived product contamination refers to the belief that a product may retain physical or symbolic traces of prior use, making it seem undesirable or unsafe (Meng & Leary, 2021). Because recycled products originate from discarded materials, they may evoke associations with dirtiness or pollution, thereby reducing purchase intentions (D’Aniello & Donato, 2025). More broadly, research suggests that contamination-related perceptions can hinder the adoption of sustainable and circular products, and that consumer responses to recycled materials are influenced by product design and communication cues (Acuti et al., 2022; Polyportis et al., 2024). Yet how these perceptions interact with perceived resource scarcity remains underexplored. This issue is theoretically important because scarcity may either intensify or attenuate the negative influence of contamination on consumer preference for recycled products.

Against this background, the present research addresses three questions. First, does perceived resource scarcity increase consumers’ preference for recycled products? Second, does holistic thinking mediate this relationship? Third, does perceived product contamination moderate this effect? To answer these questions, we conducted four studies that build on one another. Studies 1a and 1b establish the basic effect and examine its product specificity by testing whether it emerges for recycled rather than conventional products. Study 2 then advances the theorizing by identifying holistic thinking as the underlying mechanism. Finally, Study 3 extends this account by examining perceived product contamination as a boundary condition. Together, these studies provide a coherent test of the effect, its process, and its limiting condition.

## 2. Literature Review and Hypothesis Development

### 2.1. Resource Scarcity and Sustainable Consumption

Resource scarcity refers to individuals’ perceived insufficiency of available resources to meet their current needs or goals (Cannon et al., 2019). Such perceptions may arise from enduring conditions, such as chronic financial hardship or material deprivation (Hamilton et al., 2019), or from temporary situations in which resources feel insufficient to accomplish immediate objectives (Goldsmith et al., 2020; Sharma & Alter, 2012). Recent research further suggests that scarcity is not merely a structural constraint but also a psychological state that systematically shapes attention, preferences, and decision strategies (Saikia & Rahman, 2025).

Existing studies have documented that short-term resource scarcity can substantially alter consumers’ decision-making processes (Borsting et al., 2024). When resources feel constrained, consumers tend to make faster and more adaptive choices, often adjusting the criteria they use to evaluate products. For example, scarcity can reduce reliance on price as a signal of quality, prompting consumers to seek alternative cues when forming evaluations (Park et al., 2020). Scarcity has also been shown to increase preference for scarce or less accessible options, as such goods are perceived as valuable opportunities that may soon disappear (Sharma & Alter, 2012). Related work further indicates that scarcity can heighten attraction to niche or unconventional products, which may signal uniqueness or distinctiveness (Gong et al., 2021), as well as to nostalgic or anthropomorphized brands that provide emotional comfort under conditions of uncertainty (Goldsmith et al., 2020).

Despite this growing body of research, the implications of resource scarcity for sustainable consumption remain theoretically mixed. On the one hand, scarcity can heighten awareness of limited resources and motivate conservation-oriented behaviors, such as reducing waste, reusing existing products, or extending product lifecycles (Sachdeva & Zhao, 2021). Scarcity may also activate a sense of collective responsibility, encouraging individuals to consider broader societal outcomes and to support sustainability-oriented practices (Civai et al., 2024). From this perspective, resource scarcity appears to align naturally with the goals of sustainable consumption.

On the other hand, resource scarcity has also been shown to undermine green consumption. Scarcity often induces stress, anxiety, and reduced self-efficacy, which can diminish consumers’ willingness to engage in pro-environmental behaviors (Berthold et al., 2023). In addition, because many green products are perceived as more expensive or as requiring longer-term trade-offs, resource-constrained consumers may favor cheaper or immediately functional alternatives (Zhang et al., 2023). Consistent with the scarcity literature, resource constraints can also narrow consumers’ focus to pressing, short-term needs, thereby reducing sensitivity to delayed environmental benefits (Sharma & Alter, 2012).

Taken together, these contradictory findings suggest that the relationship between resource scarcity and sustainable consumption is not uniform. Rather than exerting a simple positive or negative effect, resource scarcity appears to shape sustainable consumption contingent on how consumers cognitively interpret sustainable options. Some sustainable products primarily emphasize long-term environmental benefits and abstract moral considerations, whereas others highlight immediate symbolic meaning, creativity, or transformation. This distinction implies that the psychological processes triggered by resource scarcity may differentially influence consumer responses across types of sustainable products.

Building on this insight, the present research focuses on recycled products, a distinct category of sustainable goods that foreground transformation and creative reuse rather than delayed environmental payoffs. By examining how short-term resource scarcity influences consumers’ responses to recycled products, this study aims to clarify when and how scarcity can promote sustainable consumption, and to identify the underlying psychological mechanisms driving this effect.

### 2.2. Resource Scarcity and Recycled Products

Recycled products are items made from previously used materials or objects that are converted into products with a new purpose, thereby extending the use of existing resources (Bridgens et al., 2018; Kamleitner et al., 2019). In the present research, we focus on recycled products whose material origin or prior use is communicated to consumers through product information, so that they can recognize that the product did not originate in its current form. For example, product descriptions or advertisements may indicate that a bag is made from used advertising banners or that a chair is repurposed from old wooden pallets. This feature makes recycled products different from many other sustainable products, because consumer evaluation is shaped not only by the product’s current function, but also by awareness of the material history from which it emerged (Hemonnet-Goujot et al., 2022; Oueslati et al., 2026). In other words, responses to recycled products depend in part on whether consumers regard the conversion of old materials into new offerings as worthwhile and valuable rather than merely unconventional.

Perceived resource scarcity may influence consumers’ evaluations of recycled products because the value of such products is inherently grounded in the continued use of existing materials rather than the reliance on entirely new inputs (Tang et al., 2022). When resources are perceived as limited, consumers are more likely to value options that make use of what is already available and generate new value from it. From this perspective, recycled products may become more attractive under conditions of scarcity, as they reflect both the continued utilization of existing resources and the transformation of old materials into new forms of value (Griskevicius et al., 2013; Salerno & Escoe, 2020). This reasoning is consistent with prior research showing that scarcity increases sensitivity to how resources are used and enhances the evaluation of options that emphasize efficiency, conservation, and resource-oriented value (Cannon et al., 2019; Hamilton et al., 2019; Saikia & Rahman, 2025).

In addition, research on recycled and upcycled products suggests that consumer responses are shaped not only by whether materials are non-new, but also by whether consumers perceive value in the transformation process that converts these materials into marketable products (Magnier et al., 2019). Taken together, we propose that perceived resource scarcity may increase consumer preference for recycled products by strengthening appreciation for products that derive value from materials already at hand.

Based on this reasoning, we propose a cautious hypothesis:

**H1.**  *Perceived resource scarcity increases consumer preference for recycled products.*

### 2.3. The Mediating Role of Holistic Thinking

To understand how perceived resource scarcity shapes responses to recycled products, it is important to consider the cognitive processes involved in evaluating products with layered and potentially conflicting meanings.

Recycled products are fundamentally different from conventional new products in that they embody a transformation from a prior identity to a current functional form. Evaluating such products therefore requires consumers to integrate information across time (past vs. present), function (original vs. current use), and meaning (waste vs. value) (Hemonnet-Goujot et al., 2022). As a result, judgments of recycled products often depend less on any single attribute than on whether consumers can interpret these elements as parts of a meaningful whole. This evaluative demand makes holistic thinking especially relevant. Recent research characterizes holistic thinking as a tendency to attend to relationships and the coexistence of apparently inconsistent meanings, rather than treating each cue as fully independent (Chen et al., 2022; Santos et al., 2025). Evidence from consumer judgment research similarly suggests that analytic–holistic tendencies shape evaluations when multiple cues must be processed jointly (Beekman & Seo, 2024; Dennin et al., 2022). In the case of recycled products, this processing style is particularly important because positive and negative meanings often coexist and must be interpreted together.

At first glance, proposing holistic thinking may seem inconsistent with classic scarcity research, which emphasizes narrowed cognitive bandwidth under constraint (Mani et al., 2013; Shah et al., 2012). However, these perspectives need not be contradictory. Scarcity-related narrowing primarily concerns which information become focal, whereas holistic thinking concerns how focal information is interpreted. Recent reviews likewise suggest that scarcity does not produce a single, uniform pattern of cognition or behavior, but often generates different responses depending on the demands of the decision context and the structure of the task (Civai et al., 2024). In the context of recycled products, consumers are unlikely to arrive at a stable judgment by relying on one isolated cue, because these products simultaneously convey prior use, transformation, and renewed value. Under such conditions, perceived resource scarcity may narrow attention toward resource-related concerns while also making a more relational and context-sensitive interpretation more likely.

This logic suggests that scarcity does not generally promote holistic thinking across all judgments. Rather, it is more likely to do so in contexts where consumers must make sense of competing meanings tied to limited resources. Recycled products represent such a context. When consumers are concerned with whether value can still be recognized in something previously discarded, they may become more likely to interpret prior use and current form as connected rather than contradictory. This relational mode of interpretation allows recycled products to be seen not merely as waste-derived objects, but as meaningful outcomes of resource reuse (Kamleitner et al., 2019).

Accordingly, perceived resource scarcity may increase consumer preference for recycled products indirectly by promoting holistic thinking. When consumers adopt a more relational and context-sensitive mode of interpretation, they become more likely to appreciate the transformation embedded in recycled products and to integrate their competing meanings into a coherent positive evaluation. In this sense, the present framework extends classic scarcity research by suggesting that scarcity may influence not only which information becomes focal, but also how focal information is interpreted in product categories characterized by multiple and potentially conflicting meanings.

Based on this reasoning, we propose the following hypothesis:

**H2.**  *Holistic thinking mediates the relationship between perceived resource scarcity and consumer preference for recycled products.*

### 2.4. The Moderating Effect of Perceived Product Contamination

Although products made from recycled or reused materials are often promoted as environmentally beneficial, consumers do not always respond to them positively. One important reason is perceived product contamination. Prior research shows that recycled or reused materials can evoke feelings of dirtiness or impurity, which in turn undermine consumers’ evaluations and purchase intentions, even when no objective health risk is present (Gupta & Coskun, 2021). This concern is particularly salient in sustainable consumption contexts, where consumers tend to show reluctance toward second-hand or reused products due to contamination-related beliefs (Clube & Tennant, 2020). Importantly, such concerns are not limited to visibly used goods but also apply to products made from recycled materials, such as recycled plastics, which have been shown to reduce consumers’ willingness to purchase (Meng & Leary, 2021).

Perceived product contamination arises primarily from associative processes. Consumers often form rapid and automatic judgments by linking a product to its source or prior usage, regardless of whether actual contamination exists (Morales & Fitzsimons, 2007). Once a product is mentally associated with a potentially contaminating origin, these associations can be easily reactivated during evaluation, leading to persistent negative reactions (Polyportis et al., 2024). Over time, such associative memories shape consumer judgments and discourage engagement with products perceived as recycled or reused.

These concerns are especially relevant for recycled products, whose defining feature is their connection to a prior life. Because recycled products explicitly retain traces of their original form or function, consumers may spontaneously associate them with prior environments or uses that evoke contamination concerns. For example, a chair made from industrial scrap materials may remind consumers of factories or waste sites, which can reduce its perceived attractiveness (Magnier et al., 2019). Even when consumers recognize the creativity or sustainability of repurposing, contamination-related associations may dominate their evaluations and weaken purchase intentions. Consistent with this view, research shows that consumers tend to avoid products believed to have been in contact with contaminants, even when the risk is purely symbolic rather than real (Baek & Oh, 2021).

Perceived product contamination also has important implications for how consumers respond to resource scarcity. When contamination concerns are high, negative associations with a product’s origin may override the motivational effects of scarcity (Gupta & Coskun, 2021). Consequently, consumers experiencing resource scarcity are unlikely to exhibit significantly different purchase intentions compared to those who do not perceive scarcity. In contrast, when perceived product contamination is low, the symbolic narrative of transformation embedded in recycled products may become more salient. Under these conditions, perceived resource scarcity can heighten appreciation of products that reflect resourcefulness and meaningful transformation, thereby increasing purchase intentions.

Taken together, these arguments suggest that perceived product contamination constrains the extent to which perceived resource scarcity increases consumers’ willingness to purchase recycled products. Accordingly, we propose the following hypothesis.

**H3.**  *Perceived product contamination moderates the relationship between perceived resource scarcity and consumer preference for recycled products, such that the positive effect of perceived resource scarcity is attenuated when perceived contamination is high.*

## 3. Overview of the Research

**Figure 1:**
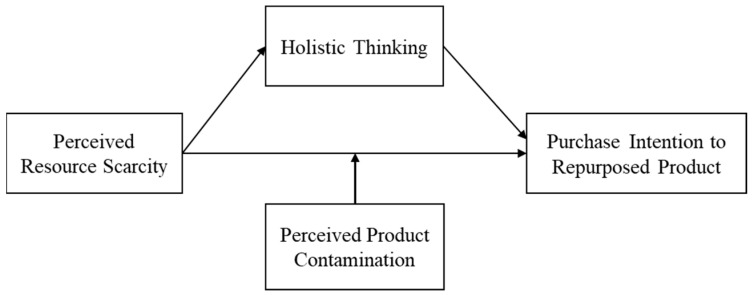
Conceptual model.

To examine how perceived resource scarcity influences consumer responses to recycled products, we conducted four experiments organized in a progressive sequence (see Figure 1). Study 1a establishes the basic effect of perceived resource scarcity on consumer interest in recycled products. Study 1b builds on this finding by testing whether the effect is specific to recycled rather than conventional products. Study 2 then examines the mediating role of holistic thinking to explain why this effect occurs. Finally, Study 3 extends the framework by testing whether perceived product contamination serves as a boundary condition. Together, these experiments provide a coherent and systematic test of the proposed theoretical framework by establishing the effect, identifying its underlying mechanism, and clarifying the condition under which it is more or less likely to emerge.

## 4. Study 1

Study 1 aims to establish the basic relationship between perceived resource scarcity and consumer interest in recycled products, and to examine whether this effect is specific to recycled rather than conventional products. Study 1a provides an initial test by examining consumers’ responses to repurposed products under perceived resource scarcity. However, focusing on repurposed products alone does not allow us to determine whether the observed pattern reflects a general response to scarcity or one that is specific to products with sustainability and transformation cues. To address this issue, Study 1b introduces ordinary products as a comparison, thereby clarifying whether the effect is specific to recycled products rather than a general tendency under perceived resource scarcity.

### 4.1. Study 1a

#### 4.1.1. Materials and Methods

114 participants (54.4% female; age: *M* = 22.77, *SD* = 2.17) were recruited from a university and randomly assigned to two conditions. The key distinction between the two conditions was the manipulation of resource scarcity. To achieve this, a recall task was used to activate specific psychological states. In the resource scarcity condition, participants were asked to recall and describe a recent experience in which they faced limitations or constraints related to resources. In contrast, participants in the non-scarcity condition were instructed to recall and describe a recent routine activity (Roux et al., 2015).

Afterward, participants read information about a recycled backpack, made from a car airbag and transformed into a comfortable, durable product (Kamleitner et al., 2019; see S1 in the Supplementary Materials). They then indicated their purchase intentions (1 = Extremely would not, 7 = Extremely would). To evaluate whether resource scarcity had been effectively activated, participants rated their perceived scarcity using items such as “I feel my resources are scarce,” “I don’t have enough resources,” and “I need to seek more resources” (1 = Completely disagree, 7 = Completely agree). Finally, participants’ gender and age, was also collected.

#### 4.1.2. Manipulation Checks

Participants reported higher perceived resource scarcity in the resource scarcity condition (*M* = 5.56, *SD* = 1.20) compared to the non-scarcity condition (*M* = 3.74, *SD* = 1.47; *t*(112) = 7.25, *p* < 0.001). This finding confirmed that the recall task used in the study was effective in inducing a sense of resource scarcity.

#### 4.1.3. Purchase Intention

Participants who experienced resource scarcity (*M* = 3.67, *SD* = 1.56) demonstrated significantly higher purchase intentions for recycled products compared to those who did not experience resource scarcity (*M* = 3.12, *SD* = 1.32; *t*(112) = 2.01, *p* = 0.047, Cohen’s *d* = 0.38, 95% *CI*: [0.01, 1.08]; see Figure 1). This finding supports H1, suggesting that resource scarcity positively influences consumers’ willingness to purchase recycled products.

### 4.2. Study 1b

#### 4.2.1. Materials and Methods

115 participants (65.2% female; age: *M* = 23.32, *SD* = 2.55) were recruited from a university and randomly assigned to two conditions. The recall task from Study 1a was used to manipulate resource scarcity. Subsequently, participants reviewed product information for a standard backpack (see S1 in the Supplementary Materials) and indicated their purchase intentions (1 = Extremely would not, 7 = Extremely would) as well as their perceived resource scarcity. Finally, participants’ gender and age were collected.

#### 4.2.2. Manipulation Checks

Participants reported higher perceived resource scarcity in the resource scarcity condition (*M* = 5.51, *SD* = 1.01) compared to the non-scarcity condition (*M* = 4.05, *SD* = 1.52; *t*(113) = 6.04, *p* < 0.001, Cohen’s *d* = 1.13, 95% *CI*: [0.98, 1.93]). This demonstrated that the recall task effectively activated perceptions of resource scarcity.

#### 4.2.3. Purchase Intention

**Figure 2:**
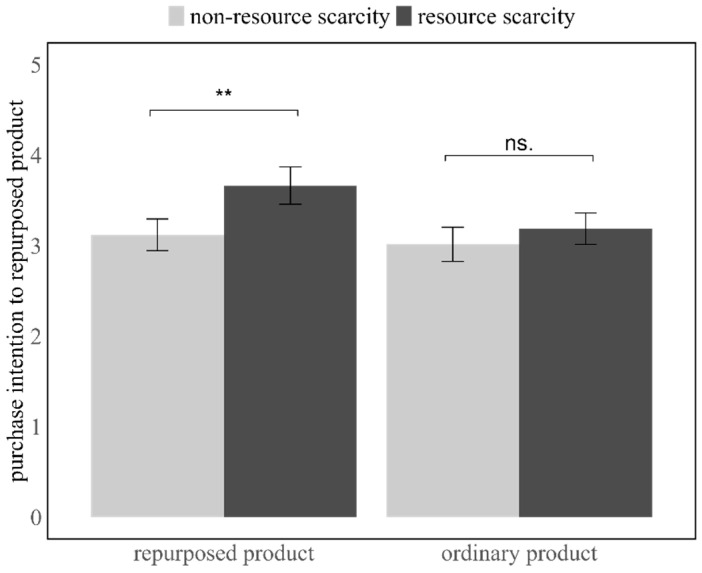
Participants exhibited higher purchase intentions for recycled products under the resource scarcity condition compared to the non-scarcity condition, while no such difference was observed for ordinary products. Error bars indicate ±1 standard error. ** denotes *p* < 0.05; ns denotes *p* > 0.05.

Participants’ purchase intention did not differ significantly between participants in the non-scarcity condition (*M* = 3.02, *SD* = 1.43) and those in the resource scarcity condition (*M* = 3.19, *SD* = 1.32; *t*(113) = 0.67, *p* = 0.50, Cohen’s *d* = 0.13, 95% *CI*: [−0.34, 0.68]; see Figure 2). This finding suggests that resource scarcity did not affect participants’ intentions to purchase the ordinary product.

### 4.3. Discussion

Taken together, the results of Studies 1a and 1b provide converging evidence for a product-specific effect of perceived resource scarcity. While perceived resource scarcity significantly increased consumers’ purchase intentions toward recycled products, it did not exert a comparable influence on ordinary products. This pattern suggests that the effect of scarcity is not general but contingent on product characteristics, particularly those related to sustainability and transformation cues.

These findings extend prior research on scarcity by showing that its influence on consumption is shaped by the symbolic and narrative meanings embedded in products. Rather than uniformly narrowing attention to immediate functional concerns, perceived resource scarcity can heighten sensitivity to products that embody resourcefulness, creativity, and meaningful transformation. In this way, recycled products appear to offer psychological value that resonates with consumers’ motivational states under scarcity.

## 5. Study 2

Building on the findings of Study 1, Study 2 examines the psychological mechanism underlying this relationship by testing whether holistic thinking mediates the effect of perceived resource scarcity on consumer interest in recycled products. To strengthen the generalizability of the proposed process, this study adopts a different manipulation of perceived resource scarcity and uses alternative recycled product materials. In this way, Study 2 advances the theorizing by explaining why perceived resource scarcity may increase preference for recycled products.

### 5.1. Materials and Methods

121 participants (60.3% female; age: *M* = 32.36, *SD* = 8.55) were recruited through the Credamo website and randomly assigned to two conditions. Resource scarcity was manipulated by having participants view different sets of images (Rodeheffer et al., 2012). In the resource scarcity condition, participants viewed images depicting scenarios related to scarcity, such as water shortages, personal financial struggles, and food shortages. In contrast, participants in the non-scarcity condition viewed neutral, everyday images, including scenes from parks, busy streets, and grocery stores (see S2 in the Supplementary Materials).

Study 2 used a real sustainable fashion brand, HowBottle, as the experimental material. The featured product was a reversible fisherman hat made from 2.7 m^2^ of recycled fishing nets and seven recycled plastic bottles (information sourced from the official website) (see S2 in the Supplementary Materials). Participants reported their purchase intentions, perceived resource scarcity, and holistic thinking after reviewing the product information. Specifically, the holistic thinking scale included statements such as: “I believe everything is interconnected,” “Nothing is completely unrelated,” “Every phenomenon has many causes, even if some of those causes are unknown,” “Focusing on the whole is more important than focusing on details,” and “The whole is greater than the sum of its parts” (1 = Completely disagree, 7 = Completely agree). These items were adapted from previous research (Choi et al., 2007).

We also measured participants’ sense of control (Cutright et al., 2013), perceived independence, perceived interdependence, relative deprivation (Smith et al., 2012) (see S4 in the Supplementary Materials) and demographic information.

### 5.2. Manipulation Checks

Participants reported higher perceived resource scarcity in the resource scarcity condition (M = 5.47, SD = 1.16) compared to the non-scarcity condition (*M* = 4.15, *SD* = 1.66; *t*(119) = 5.11, *p* < 0.001, Cohen’s *d* = 0.92, 95% *CI*: [0.81, 1.84]). The result confirms that the image observation task effectively induced resource scarcity.

### 5.3. Purchase Intentions

Participants who experienced resource scarcity (*M* = 5.50, *SD* = 1.01) had significantly higher purchase intentions for recycled products compared to the non-scarcity condition (*M* = 5.06, *SD* = 1.18; *t*(119) = 2.19, *p* = 0.03, Cohen’s *d* = 0.40, 95% *CI*: [0.04, 0.83]). This finding further supported H1.

### 5.4. Mediation Effect of Holistic Thinking

**Figure 3:**
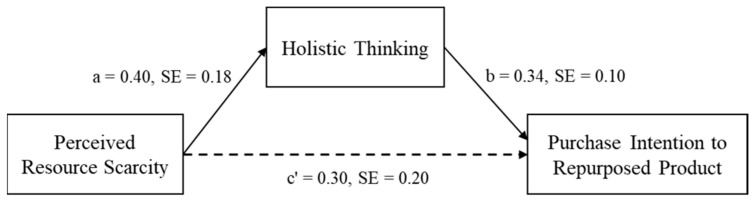
Path diagram. Note: A solid line indicates a significant path (*p* < 0.05).

To examine the mediation effect of holistic thinking, we used the Bootstrap method proposed by Hayes (2009) (Model 4; with 5000 bootstrap samples and a 95% confidence interval). The results showed that holistic thinking significantly mediated the relationship between perceived resource scarcity and purchase intention (*β* = 0.13, *SE* = 0.08, *p* = 0.04, 95 *CI*: [0.01, 0.31]; see Figure 3). Examining the individual paths, experiencing resource scarcity significantly predicted holistic thinking (*β* = 0.40, *SE* = 0.18, *p* = 0.03, 95 *CI*: [0.04, 0.75]), and, in turn, holistic thinking significantly predicted purchase intention (*β* = 0.34, *SE* = 0.10, *p* = 0.003, 95 *CI*: [0.11, 0.55]). In other words, participants under resource scarcity exhibited more holistic thinking than those who were not, which increased their purchase intentions. This finding supported H2. Furthermore, the mediating effects of sense of control, perceived independence, perceived interdependence and relative deprivation were not significant (all *ps* > 0.05), suggesting that these factors did not contribute to the relationship between resource scarcity and purchase intentions.

### 5.5. Discussion

The results of Study 2 provide insight into how perceived resource scarcity affects consumer preferences for recycled products. The findings indicate that holistic thinking is a key factor in this process. When consumers perceive limited resources, they tend to consider the connections between a product’s past and present use more carefully, rather than focusing solely on immediate or practical aspects. This allows them to appreciate the value of transformation and reuse. Tests for alternative explanations, including sense of control, relative deprivation, and self-construal, showed no significant effects, suggesting that the observed pattern is primarily due to changes in cognitive processing rather than motivational or identity-related factors.

## 6. Study 3

While Studies 1 and 2 establish the basic effect and its underlying mechanism, Study 3 examines an important boundary condition of this relationship. Specifically, it tests whether perceived product contamination moderates the influence of perceived resource scarcity on recycled product preference. Based on our theorizing, we expected that perceived resource scarcity would increase purchase intentions for products perceived as low in contamination, but not for those perceived as high in contamination. To test this prediction, we manipulated contamination level and introduced a new set of product stimuli. Thus, Study 3 extends the theoretical account by clarifying when the positive effect of perceived resource scarcity on recycled product preference is more or less likely to emerge.

### 6.1. Pretest

50 participants (62% female; age: *M* = 31.02, *SD* = 9.72) were recruited through the Credamo website and randomly assigned to two conditions. The preliminary study followed the procedures from Kamleitner et al. (2019) and used wallets made from mosquito nets as the experimental stimulus. In the high product contamination condition, the wallet was made from worn and soiled mosquito nets, while in the low product contamination condition, the wallet was crafted from clean, used mosquito nets (see S3 in the Supplementary Materials).

Participants’ perceptions of product contamination were assessed using items such as: “How dirty do you think the product is? (1 = Not dirty, 7 = Very dirty),” “How unsanitary do you think the product is? (1 = Very sanitary, 7 = Very unsanitary),” and “To what extent do you think the product is contaminated? (1 = Not contaminated, 7 = Very contaminated)” (Argo et al., 2006).

Participants in the high product contamination condition reported higher levels of perceived product contamination (*M* = 3.81, *SD* = 1.59) compared to the low product contamination condition (*M* = 2.61, *SD* = 1.37; *t*(48) = 2.86, *p* = 0.006, Cohen’s *d* = 0.81, 95% *CI*: [0.36, 2.04]). This finding confirms that the experimental materials successfully created a clear contrast between high and low product contamination conditions.

### 6.2. Materials and Methods

245 participants (66.5% female; age: *M* = 30.21, *SD* = 7.09) were recruited via the Credamo platform. We employed a 2 (perceived resource scarcity: scarcity vs. non-scarcity) × 2 (perceived product contamination: high vs. low) between-subjects design. To manipulate resource scarcity, the procedure from Study 1a was replicated. Afterward, participants reviewed product information about a recycled wallet (the same material used in the pretest) and indicated their purchase intention (1 = Definitely would not, 7 = Definitely would), as well as their perceived resource scarcity and perceived product contamination. Additionally, we measured participants’ sense of control (Cutright, 2012), current economic status (Griskevicius et al., 2013) and childhood economic status (Rindfleisch et al., 1997). Finally, demographic information was collected.

### 6.3. Manipulation Checks

Participants in the resource scarcity condition (*M* = 4.92, *SD* = 1.71) reported higher levels of perceived resource scarcity compared to the non-scarcity condition (*M* = 3.85, *SD* = 1.53; *t*(243) = 5.18, *p* < 0.001, Cohen’s *d* = 0.66, 95% *CI*: [0.67, 1.49]), demonstrating the effectiveness of the recall task in activating resource scarcity. Additionally, perceived product contamination was higher in the high product contamination condition (*M* = 3.85, *SD* = 1.56) compared to the low product contamination condition (*M* = 2.95, *SD* = 1.33; *t*(243) = 4.86, *p* < 0.001, Cohen’s *d* = 0.62, 95% *CI*: [0.53, 1.26])), confirming the efficacy of the product images in eliciting perceived contamination.

### 6.4. Purchase Intentions

The results revealed a significant main effect of perceived product contamination (*F*(1, 243) = 40.10, *p* < 0.001, *η_p_*^2^ = 0.08), as well as a significant interaction effect between perceived resource scarcity and perceived product contamination on purchase intentions (*F*(1, 241) = 7.40, *p* = 0.048, *η_p_*^2^ = 0.02). Nevertheless, the main effect of perceived resource scarcity was not significant (*F*(1, 241) = 1.15, *p* = 0.28, *η_p_*^2^ = 0.005).

**Figure 4:**
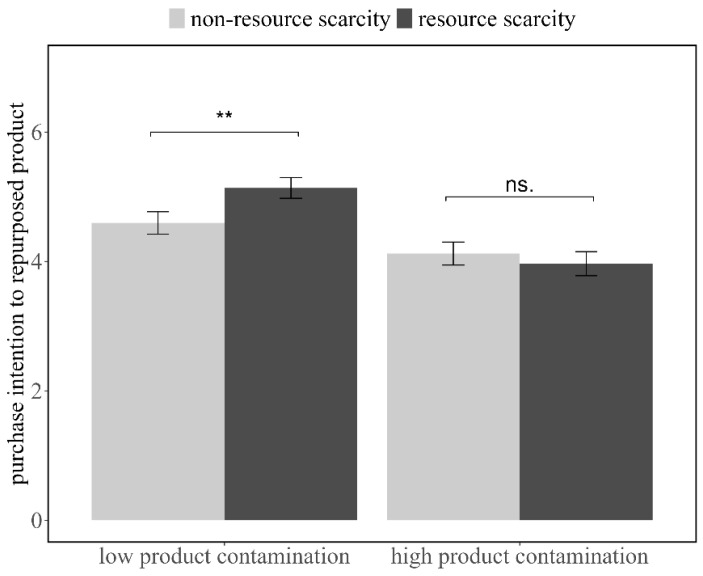
Under the low product contamination condition, participants experiencing resource scarcity exhibited higher purchase intentions compared to those in the non-scarcity condition. However, this difference was not observed in the high product contamination condition. Error bars indicate ±1 standard error. ** denotes *p* < 0.05; ns indicates *p* > 0.05.

When perceived product contamination was low, participants in the resource scarcity condition showed greater purchase intentions (*M* = 5.14, *SD* = 1.22) compared to the non-scarcity condition (*M* = 4.60, *SD* = 1.36; *t*(118) = 2.30, *p* = 0.02, Cohen’s *d* = 0.42, 95% *CI*: [0.07, 1.01]). In contrast, no significant difference in purchase intentions was observed between the resource scarcity (*M* = 3.97, *SD* = 1.44) and non-scarcity conditions (*M* = 4.12, *SD* = 1.44) when participants were exposed to high product contamination (*t*(123) = −0.61, *p* = 0.55, Cohen’s *d* = 0.10, 95% *CI*: [−0.67, 0.35]; see Figure 4).

### 6.5. Moderation Effect of Perceived Product Contamination

To test the moderating effect of perceived product contamination, we used the Bootstrap method proposed by Hayes (2009) (Model 1; with 5000 bootstrap samples and a 95% confidence interval). The results revealed a significant moderation effect (*F*(1,241) = 3.96, *p* = 0.048). Under the low product contamination condition, participants showed higher purchase intentions in the resource scarcity condition than in the non-scarcity condition (*β* = 0.54, *SE* = 0.25, *p* = 0.04, 95% *CI*: [0.05, 1.03]). However, under the high product contamination condition, participants’ purchase intentions showed no difference between the resource scarcity and non-scarcity conditions (*β* = −0.16, *SE* = 0.25, *p* = 0.52, 95% *CI*: [−0.64, 0.33]). These findings support the moderating role of perceived product contamination, thereby validating H3. Furthermore, the effects of sense of control, current economic status and childhood economic status were not significant (all *p* > 0.05), further confirming that these variables did not influence the moderation effect observed in this study.

### 6.6. Discussion

Building on the findings from Studies 1 and 2, Study 3 further examined how product contamination moderates the impact of perceived resource scarcity on consumer behavior regarding recycled products. Specifically, consumers who perceive low levels of product contamination and experience resource scarcity show a greater willingness to purchase recycled products. However, when product contamination is high, their willingness to purchase recycled products does not differ, suggesting that high contamination levels may override the influence of scarcity on consumer decision-making.

## 7. General Discussion

### 7.1. Conclusion

This research shows that perceived resource scarcity can increase consumer preference for recycled products, but that this effect is neither general nor unconditional. Rather, it emerges in a product category whose evaluation depends in part on how consumers make sense of material history and transformation. Specifically, scarcity appears to increase the appeal of recycled products by encouraging a more integrative interpretation of prior use and present value, whereas this positive effect is weakened when contamination concerns become salient. Taken together, these findings provide an integrated account of how perceived resource scarcity shapes consumer responses to recycled products by clarifying the effect itself, its underlying mechanism, and the condition under which it is constrained.

Although the observed effect sizes were generally modest, this pattern should be interpreted in light of the nature of consumer decision making, in which preferences are rarely shaped by a single factor in isolation. From a theoretical perspective, the effects are meaningful because they were directionally consistent across studies and supported under different manipulations and product contexts. From a practical perspective, the findings remain relevant because they point to low-cost and scalable ways of increasing acceptance of recycled products, such as emphasizing their resource origin and transformation in product communications. In settings where consumers repeatedly choose between recycled and conventional products, even modest shifts in evaluation may affect adoption. Overall, the present findings suggest that consumers’ perceptions of resource scarcity and their interpretations of product history jointly shape willingness to embrace recycled products.

### 7.2. Theoretical Implications

First, this research advances the literature on resource scarcity by broadening its explanatory scope. Prior research has mainly examined how perceived resource scarcity shapes consumer responses in situations involving constrained choice, immediate trade-offs, or short-term decision priorities. By contrast, the present research shows that perceived resource scarcity also matters in the evaluation of products whose value is not determined solely by current utility, but must be understood through the coexistence of favorable and unfavorable meanings. In the case of recycled products, consumers evaluate not only what the product can offer in the present, but also what prior use and subsequent transformation imply for its current worth (Hemonnet-Goujot et al., 2022; Sulaimani et al., 2025). The theoretical contribution therefore does not lie simply in applying scarcity theory to a new setting. Rather, it lies in showing that perceived resource scarcity can shape product evaluation when value depends on how consumers interpret and reconcile conflicting meanings. In this way, the present research extends the relevance of scarcity theory beyond constrained choice and into a more interpretive form of consumer evaluation (Kim et al., 2022; Hemonnet-Goujot et al., 2022; Saikia & Rahman, 2025).

Second, this research refines existing views of how perceived resource scarcity affects cognition. Much of the prior literature has emphasized the restrictive consequences of scarcity, suggesting that it narrows attention, reduces cognitive bandwidth, and heightens focus on urgent needs (Mani et al., 2013; Shah et al., 2012; Zhang et al., 2023). Although this perspective has generated important insights, it does not fully explain product evaluation in contexts where consumers must make sense of objects that carry internally contradictory meanings. The present research suggests that perceived resource scarcity may influence not only what consumers attend to, but also how they make sense of what they encounter. The role of holistic thinking is theoretically important in this regard. Its contribution is not merely that of an intervening variable. More importantly, it indicates that perceived resource scarcity may be associated with a more integrative mode of interpretation when consumers evaluate products that combine seemingly inconsistent implications within the same object (Chen et al., 2022; Santos et al., 2025; Beekman & Seo, 2024). This refines the dominant view of scarcity as uniformly constraining and offers a more differentiated account of cognitive adaptation under perceived resource constraints.

Third, this research clarifies the boundary of this process by showing that the positive influence of perceived resource scarcity depends on whether conflicting product meanings can be resolved in a favorable way. Prior research has shown that contamination concerns often undermine consumer responses to reused, recycled, and previously owned products because traces of prior use can evoke aversion and reduce desirability (Baek & Oh, 2021; Acuti et al., 2022; Polyportis et al., 2024). The present research extends this literature by showing that contamination does more than exert a direct negative influence on evaluation. It also limits whether the interpretive process associated with perceived resource scarcity can ultimately produce a positive judgment. Even when consumers become more inclined to integrate different aspects of the product, this process is less likely to support favorable evaluation when negative meanings remain dominant and difficult to absorb into a coherent understanding (D’Aniello & Donato, 2025). The contribution here is that the effect of perceived resource scarcity is shown to be conditional rather than universal. Its positive implications emerge only when product meanings remain open to reconciliation.

Taken together, these contributions provide a more integrated account of consumer evaluation under perceived resource scarcity. Rather than treating scarcity as a uniformly restrictive condition, this research shows that its consequences depend on how product meaning is formed during evaluation and on whether that meaning can be interpreted in a favorable direction. In doing so, the present research brings scarcity theory into closer dialogue with sustainable consumption research and offers a more nuanced understanding of consumer responses to recycled products.

### 7.3. Managerial Implications

This research offers several actionable implications for managers seeking to promote recycled products in a way that aligns with how consumers respond to resource scarcity. Rather than emphasizing sustainability in abstract terms, firms can improve market acceptance by carefully shaping how recycled products are framed, presented, and communicated.

First, marketers should highlight transformation rather than waste. Communications should focus on the creative process through which discarded materials are transformed into new, functional products. Emphasizing craftsmanship, design ingenuity, and purposeful reuse helps shift attention away from the product’s discarded origins and toward its meaningful transformation. This approach allows recycled products to be perceived as resourceful and innovative, rather than as inferior substitutes for new goods.

Second, firms should actively provide contamination assurance. Because perceived contamination constrains consumers’ willingness to purchase recycled products, managers should incorporate clear signals of cleanliness and quality control. Certifications, professional processing descriptions, sanitation standards, and quality guarantees can reassure consumers that recycled products meet the same standards as new products. Without such assurances, even well-designed sustainability messages may fail to translate into purchase intentions.

Third, managers should frame recycled product consumption as a smart and resourceful choice. Under conditions of resource scarcity, consumers are more responsive to signals of ingenuity and value-savviness than to moral or environmental appeals alone. Positioning recycled products as evidence of clever decision-making, efficient resource use, and practical creativity may therefore resonate more strongly than framing them solely as environmentally responsible options.

Finally, communication strategies should be tailored to product category characteristics. For product categories that are especially vulnerable to contamination inferences, such as apparel or personal items, visual and verbal cues should minimize references to prior use and instead emphasize refinement and quality control. In contrast, for categories that are less contamination-sensitive, such as home decoration products or furniture, highlighting the product’s origin story and creative transformation may be more effective. Aligning communication strategies with category-specific contamination sensitivity can improve consumer acceptance across diverse recycled product markets.

### 7.4. Limitations & Future Research

While this study enhances our understanding of how resource scarcity affects recycled product consumption, it has limitations in terms of applicability and scope.

First, the generalizability of the findings remains to be further established. Although the present studies used different materials and measurement approaches, the samples were drawn from Chinese participants. This issue is particularly relevant because holistic thinking has often been associated with East Asian cognitive styles, which means that the observed effects may partly reflect cultural tendencies toward contextual and integrative processing. Future research could therefore test the proposed framework in more diverse cultural and geographical settings and examine whether the relationship between perceived resource scarcity and recycled product preference varies across social and cultural groups (Jiang et al., 2025). In addition, more naturalistic and longitudinal designs would help assess whether the observed patterns extend to real world consumption behavior and remain stable over time.

Second, the explanatory scope of the model can be further developed. Although the present research identifies holistic thinking as the key mechanism linking perceived resource scarcity to recycled product preference, the modest effect sizes across studies suggest that this relationship is likely shaped by multiple influences. Other psychological factors may also contribute to this process. In particular, meaning seeking, identity signaling, and moral motivation may each help explain why consumers under perceived resource scarcity become more inclined to evaluate recycled products in an integrative way. When resources are perceived as limited, consumers may attach greater value to meanings related to renewal and resourcefulness, become more attentive to the identity implications of their choices, or view recycled products as a more morally appropriate response to constrained resources (Salciuviene et al., 2022). These factors may not function as explanations separate from holistic thinking, but rather as antecedent motivations that make such thinking more likely. Future research could therefore examine whether these factors exert similar or distinct influences, whether they shape holistic thinking through different pathways, and whether their effects vary across product types or consumer groups.

Third, the boundary conditions of the effect deserve further attention. Consumer responses to recycled products under perceived resource scarcity are likely influenced by a broader set of contextual factors than those examined here. For example, price may play an important role in actual consumption settings, even though it was held constant in the present studies. In addition, although participants were randomly assigned to conditions, which reduces the likelihood that pre-existing preferences systematically differed across groups, baseline interest in the focal product was not directly measured. Future research could therefore assess consumers’ prior interest in specific product categories more explicitly when examining purchase intentions. Product characteristics such as aesthetics or practicality may also shape consumers’ responses. In addition, different forms of scarcity may not operate in the same way. Scarcity involving money or material resources may direct attention toward affordability and functionality, whereas scarcity involving time or social status may heighten sensitivity to symbolic value and social meaning. Future research could further examine how these factors interact with perceived resource scarcity to shape consumer responses to recycled and previously used products.

Taken together, these directions would help build a more complete understanding of when and why perceived resource scarcity influences recycled product preference and would further clarify the motivational and contextual foundations of sustainable consumption under constraint.

## Data Availability

Data supporting reported results are available from the authors on request.

## Supplementary Materials

The following supporting information can be downloaded at: https://www.mdpi.com/article/10.3390/bs16050673/s1, Figure S1: Experimental materials for Study 1a; Figure S2: Experimental materials for Study 1b; Figure S3: Experimental materials used in Study 2 to manipulate resource scarcity; Figure S4: Experimental materials for Study 2; Figure S5: Experimental materials used in Study 3 for the high product-contamination condition; Figure S6: Experimental materials used in Study 3 for the low product-contamination condition; Table S1: Measurement scales.
