# Supplementary material for: When Scarcity Meets Sustainability: Consumer Preferences for Recycled Products
Source: Behav Sci (Basel). 2026 Apr 29;16(5):673. doi: 10.3390/bs16050673 (PMC13203598; doi:10.3390/bs16050673)
Supplement: Supplementary file 1 [file behavsci-16-00673-s001.zip › behavsci-4151857-supplementary.pdf]

S1（STUDY 1 STIMULI）

Study 1a Stimuli

Please imagine the following situation and answer the questions. You recently wanted to buy a backpack, and you happen to see this backpack, which used to be **a car airbag,** made into a comfortable and durable backpack.

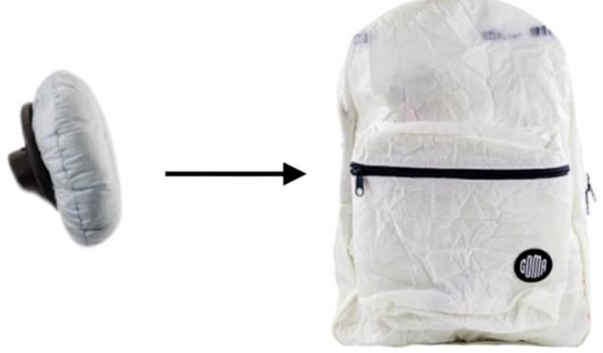

Figure S1. Experimental materials for Study 1a

Study 1b Stimuli

Please imagine the following situation and answer the questions. Imagine that you recently wanted to buy a backpack, you happen to see this backpack, it is a comfortable and durable backpack.

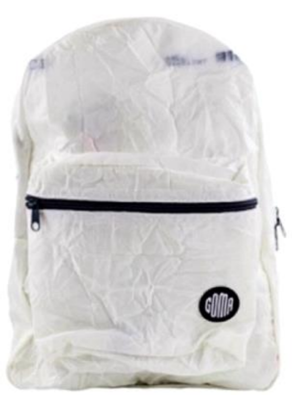

Figure S2. Experimental materials for Study 1b

S2（STUDY 2 STIMULI）

resource scarcity condition

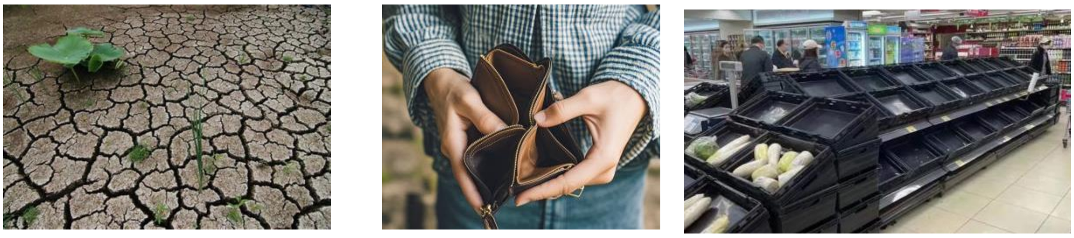

non-scarcity condition

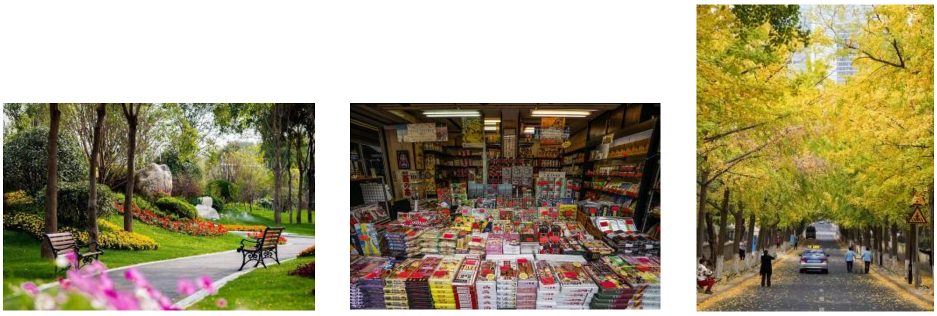

Figure S3. Experimental materials used in Study 2 to manipulate resource scarcity

Please imagine the following situation and answer the questions. You recently wanted to buy a hat, and you happen to see this hat, which was once **a mineral water bottle and a fishing net**, made into a simple and comfortable double-sided fisherman's hat.

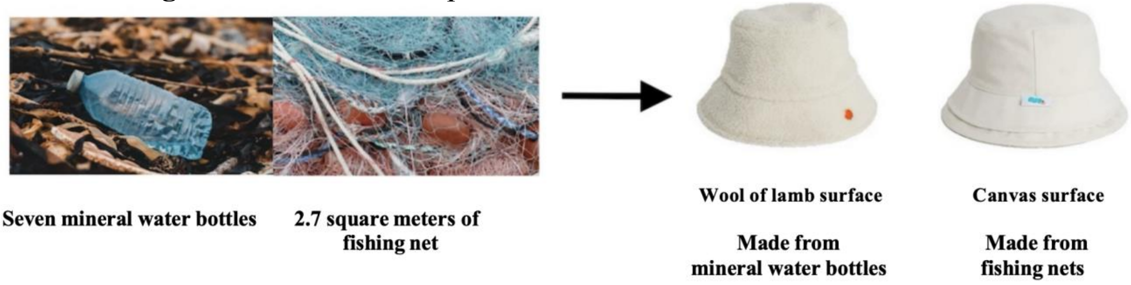

Figure S4. Experimental materials for Study 2

S3（STUDY 3 STIMULI）

High-product-contamination condition

Please imagine the following situation and answer the questions. You recently wanted to buy a wallet, and you came across this wallet, which used to be a mosquito net, made into a stylish and durable handmade wallet.

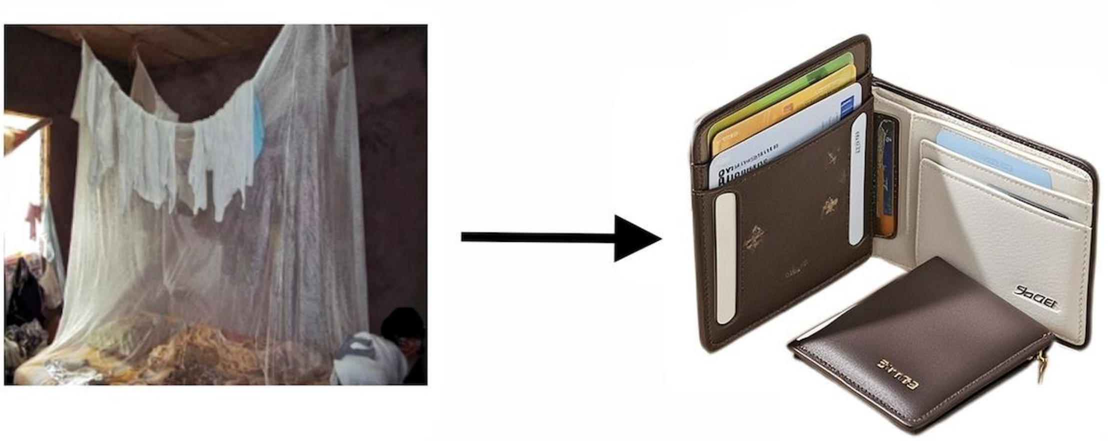

Figure S5. Experimental materials used in Study 3 for the high product-contamination condition

Low-product-contamination condition

Please imagine the following situation and answer the questions. You recently wanted to buy a wallet, and you came across this wallet, which used to be a mosquito net, made into a stylish and durable handmade wallet.

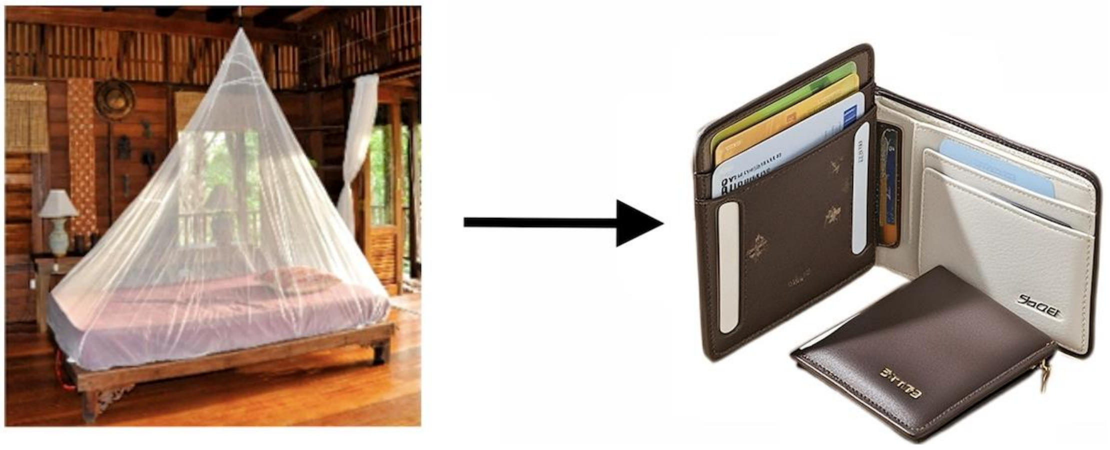

Figure S6. Experimental materials used in Study 3 for the low product-contamination condition

S4（Measurement scales)

Table S1. Measurement scales

Constructs	Items	Reference
childhood economic status	In childhood, the family has enough money to meet all kinds of needs	Rindfleisch et al., 1997
	Compared to my childhood friends, my life is more prosperous	
	I grew up in an affluent and stable community	
current economic status	I have enough money to buy what I want	Griskevicius et al., 2011
	I don't need to worry too much about paying the daily bills	
	I don't think I need to worry too much about money in the future	
sense of control	I often feel helpless in dealing with the events of my life	Cutright, 2012
	Things happen in my life that are completely out of my control	
	Accidents happen in my life that prevent me from doing what I want	
perceived interdependence	It is important for me to respect the decisions of the group	Pan & Lv, 2013
	I often feel that my own achievements are more important than my relationships with others	
	I will sacrifice my own interests for the interests of my group	
	My happiness depends on the happiness of those around me	

perceived independence	It is important to me that my personality traits are not constrained by others	Pan & Lv, 2013
	I want to be different in many ways	
	I do not feel uncomfortable when I am singled out for praise or reward	
	I prefer to be open and honest with myself rather than being misunderstood	
relative deprivation	Compared with my efforts and efforts, my life should be better than it is now	Smith et al., 2012
	I always feel that others have taken what should belong to me	
	Compared with the people around me, I am quite disadvantaged in life, work and other aspects	
	Those rich people in society are mostly made rich through dishonest means	
perceptions of product contamination	How dirty do you think the product is?	Argo et al., 2006
	How unsanitary do you think the product is?	
	To what extent do you think the product is contaminated?	
